# A Snapshot of Bayesianism

**DOI:** 10.3390/e27040448

**Published:** 2025-04-21

**Authors:** Mark A. Gannon

**Affiliations:** Independent Researcher, São Paulo 05508-090, SP, Brazil; mark.a.gannon@gmail.com

Students are told in basic probability classes that there are two main “schools” of statistics, the frequentist and the Bayesian, and that those different views of how to approach statistical inference problems arise from two different views of the meaning of probability. Practicing scientists know things are not that simple.

First, statisticians as a group cannot be divided so simply into two schools. There are other approaches, such as likelihoodism. Additionally, the schools are not mutually exclusive. All three of the invited academic editors of this special edition of *Entropy* have published work that could be classified as using a frequentist approach and work that could be classified as using a Bayesian approach. There are even articles in our publication history that report on methods that blend Bayesian and frequentist approaches to inference problems, and this is not as unusual as teachers of basic probability courses would have us believe. It is not uncommon for researchers who use one of the two major approaches to statistics in much of their work to occasionally use a concept, tool, or procedure from another “school” of statistics to solve an otherwise intractable problem.

Additionally, even among those using Bayesian approaches to parametric inference, there are differing views on how to choose prior distributions for the parameters of interest. Conjugate priors are useful for theoretical work, because they facilitate closed-form analytic results, but sometimes there is no choice of hyperparameters that can make a given conjugate prior reflect what experts expect of the distribution of the parameters. Among the statisticians with a more “subjectivist” approach, elicitation of priors is a way of translating expert knowledge into a mathematical starting point for inference. Those with a more “objectivist” approach attempt to reduce the influence of subjective choices made by human beings, with all of our flaws and biases.

Subjectivist and objectivist Bayesians adopt concepts from each other, so the division between those approaches to Bayesian inference also tends not to be clearly defined. The father of modern subjectivist Bayesian statistics, Bruno de Finetti, treated probability as a degree of belief in his work, and that view has been adopted by many Bayesians, regardless of their views on how to choose prior distributions. Objectivist Bayesians tend to think of probability as a degree of rational belief like subjectivists do, but they start with priors meant to represent “neutral” knowledge rather than an initial individual or group belief. Subjectivist Bayesians occasionally use “non-informative” priors that are defined using the methods of objectivist Bayesian inference. While the classification of approaches to statistics into “schools” and the classification of Bayesian approaches as objectivist or subjectivist do not define clear partitions, they are still useful for understanding approaches to a given problem, the differences between them, and their relative strengths and weaknesses.

The present Special Issue of *Entropy* was conceived as an attempt to provide a “snapshot” of Bayesian thought and Bayesian inference in 2024. And while 16 articles cannot do justice to the breadth and depth of the theoretical work, simulation studies, and real-world data analysis using Bayesian ideas and methods worldwide, we feel that the content of this Special Issue provides a representative sample of the wide variety of current directions in Bayesian-inference-based research and applications.

Despite the fact that *Entropy* does not usually publish “perspective” articles, the invited academic editors of this Special Issue consider the subject of the article “Statistics as a Social Activity: Attitudes toward Amalgamating Evidence” [contribution 1] sufficiently important to such an immensely broad range of activities, and the approach taken to the subject sufficiently unique, forward-thinking, and broadly applicable, that it was worth arguing for an exception to be made. That article makes this Special Issue a snapshot of much more than just Bayesianism, bringing all of statistics, science, and all the potential applications of descriptive statistics and inference into the picture by treating some of the most fundamental issues in data analysis.

Among the major applications of statistics in the mid-2020s is “machine learning”, where “supervised machine learning”—the variety that is appearing frequently in news reports—refers to algorithms for making various types of regression models, with classification being an important subclass of regression models with small, discrete ranges. The article entitled “Probabilistic Nearest Neighbors Classification” [contribution 2] takes a Bayesian approach to delving into a widely used classification technique, developing a theoretical understanding of its computational complexity, and using that knowledge to produce a practical real-world result: a new model that performs as well for classification tasks as the previous algorithm, but with more manageable computational cost. Statisticians naturally have important roles to play in machine learning, and this article is an example of advances in machine learning algorithms made possible by a Bayesian approach.

One of the editors of the present Special Issue is the creator of the idea behind the Full Bayesian Significance Test (FBST) [1,2,3], a Bayesian replacement for the familiar and oft-abused Neyman–Pearson hypothesis tests and Fisher significance tests, with the “*e*-value” playing a similar role to the more familiar *p*-value, a measure of the compatibility of observed data with a null hypothesis. The FBST has been used in a variety of fields, and researchers around the world have analyzed, explored, and extended it with further theoretical work (see, for example [4,5,6,7,8,9,10,11], and references cited in section 4 of [3]). A common issue in parametric statistics is how to handle inferences about some of a given model’s parameters in the presence of “nuisance parameters”, the name given to the model’s other parameters—the ones about which inferences are *not* being drawn. As its title suggests, the article “Objective Priors for Invariant e-Values in the Presence of Nuisance Parameters” [contribution 3] takes an objectivist-Bayesian approach to exploring properties of the FBST in cases with nuisance parameters. Such work is important because advances in our understanding of existing inferential tools like the FBST and their properties contribute to the maturation of those tools and potentially guide the development of new ones.

Poisson distributions are widely used to model counts of infrequent occurrences in fixed time intervals. In 1960, the possibility of an observer not noticing a single occurrence in a given time interval was considered, allowing for an interval in which there was actually one occurrence being recorded as having none, leading to the “misrecorded Poisson distribution” or “Cohen–Poisson distribution”, which is studied further in the article “Linear Bayesian Estimation of Misrecorded Poisson Distribution” [contribution 4] in the present Special Issue. The authors propose and show the advantages of a linear Bayesian estimation procedure for the parameters of the misrecorded Poisson distribution, including demonstrating, via numerical studies and specific examples using real-world data, that the performance of the linear-Bayesian approach to estimating those parameters is superior to that of maximum-likelihood estimation. This article represents a Bayesian approach to one way of handling the possibility of misrecorded count data.

Because the elicitation of expert knowledge frequently does not yield a unique possible prior distribution, much Bayesian research, especially in the last two decades of the 20th Century and the first decade of the 21st Century, focused on the issue of robustness, defined as the insensitivity of inferential results to the inputs of the inferential process, including the choice of a prior. One-sample and two-sample *t*-tests have been used for over a century, with both frequentist and Bayesian versions devised in the early 20th Century. “An Objective and Robust Bayes Factor for the Hypothesis Test One Sample and Two Population Means” [contribution 5] takes an objectivist-Bayesian approach to two classic hypothesis testing problems. The authors propose an objective and robust Bayes factor and use it to analyze real-world data and show the finite-sample consistency of these tools. The authors also develop a modified version of the Bayesian information criterion for comparing sample means and find that, in practice, their approaches to two-sample comparisons tend to yield similar results to each other, including support for the null hypothesis in the case of data generated from distributions with equal means and variances. This article represents theoretical statistics work to develop tools for researchers in a wide range of fields of study, the continued updating of objectivist Bayesian methods of statistical inference, and specifically, the refinement of robust-analysis objectivist Bayesianism.

“On the Nuisance Parameter Elimination Principle in Hypothesis Testing” [contribution 6] treats inference in the presence of nuisance parameters in the specific case of hypothesis testing. It does so by developing a form of Berger and Wolpert’s non-informative nuisance parameter principle (NNPP) that is appropriate for hypothesis tests, exploring the compatibility of that form of the principle with both Bayesian and non-Bayesian views and methods, and treating a specific example by showing that a class of hypothesis tests that mix elements from the Bayesian and frequentist paradigms, proposed and developed by Pereira and collaborators [12,13], adheres to the NNPP when the sample space is discrete and the parameter space finite and variation-independent. The authors further show that the adherence of those tests to the NNPP can facilitate calculations and reduce the computing cost of performing the mixed-paradigm tests. In this article, we can see a Bayesian approach to a fundamental issue in theoretical statistics yielding practical results, facilitating the real-world use of a relatively new inferential tool.

“Stochastic Volatility Models with Skewness Selection” [contribution 7] extends the stochastic volatility models that have been successful at modeling asset returns in financial time series, doing so in a way that permits dynamic skewness but does not require it. The authors employ a data-driven sparsity scheme for their model’s parameters, similar in concept to the shrinkage in LASSO regression, but based on a Bayesian approach, that chooses from three possibilities based on the data: dynamic skewness, static skewness, and zero skewness. They then apply their new models to financial time series, showing examples that result in dynamic skewness and zero skewness, with implications for some of the biggest issues in econometrics, finance, and even monetary policy. This article is an example of a Bayesian approach to asset-return modeling yielding a more flexible extension of previously existing models that can deepen our understanding of the behavior of asset returns and financial markets.

Extreme value theory seeks to provide inferences for rare events, that is, those found in the tails of probability distributions. “Peaks-over-threshold” approaches use the subset of points that are peaks of periods of a time series that are above a threshold value as a way of selecting the most extreme events in the available time series. Theoretical results such as the Pickands–Balkema–De Haan Theorem then allow the peaks-over-threshold subset to be used for inferences about very rare events, and this can have important applications in risk assessment for extreme climate and weather events, financial market crashes, and other situations in which rare and extreme events can have wide-ranging impacts. “Bayesian Non-Parametric Inference for Multivariate Peaks-over-Threshold Models” [contribution 8] starts with the Pareto process, a generalization to infinite dimensionality of the generalized Pareto distribution that arises in univariate peaks-over-threshold extreme value analysis, shows how the Pareto process can be decomposed into radial and angular components, with the angular component containing information about the dependence structure of the random vector, and proposes a novel non-parametric Bayesian approach to modeling the angular component. The technique is applied to synthetic data and to using real-world integrated vapor transport data to estimate the extremal dependence structure for a measure of water vapor flow in the atmosphere. The advances reported in the article are set up as steps on the way to detailed modeling of atmospheric events that lead to storm activity. The article brings the important area of extreme value theory and its applications into our snapshot of the applications of Bayesian methods in 2024.

“Bayesian Spatio-Temporal Modeling of the Dynamics of COVID-19 Deaths in Peru” [contribution 9] brings an example of spatio-temporal data analysis and Bayesian spatio-temporal modeling, applied to understanding public health data on COVID-19 in Peru. The study models the count of COVID-19 deaths as the response variable, with covariates including the total number of COVID-19 cases and the counts of first, second, third, and fourth doses of vaccines administered, all measured 14 days before the corresponding COVID-19 death count. Their analysis of Peruvian public health data shows, for example, that higher coverage of the first two doses of COVID-19 vaccines reduced the number of deaths in the period studied, with third-dose coverage having an even stronger effect. This article demonstrates the flexibility of Bayesian methodologies in modeling real-world data, particularly spatio-temporal data. The authors show how these methods allow them to reach conclusions that could inform decisions by public health authorities and legislators regarding health policy and interventions.

“A Metric Based on the Efficient Determination Criterion” [contribution 10] uses a generalization of the Bayesian information criterion (BIC) to extend a BIC-based metric that has been indispensable for estimating minimal partitions in research involving partition Markov models, including models of viral genetic sequences, Huffman coding, and data compression. The BIC-based metric allows for strong consistency in estimating a minimal partition, which in turn allows for more efficient estimation of the model’s transition probabilities. The authors introduce the efficient determination criterion (EDC), a generalization of the BIC, and prove that it can serve as the basis of a metric similar to the BIC-based metric, but with different possible terms to penalize higher numbers of model parameters. They show that strong consistency can be maintained with a penalty term of the form ln(ln(n)), where *n* is the number of parameters in the model. The authors then apply their new metric to a specific application, modeling three genetic sequences of dengue virus type 3, the return of which to Brazil could have serious public health implications, as the population is expected not to have immunity due to the length of time the virus has been nearly absent.

Copulas are used to model the dependence between random variables. “Likelihood Inference for Factor Copula Models with Asymmetric Tail Dependence” [contribution 11] addresses a limitation of copulas and proposes a Bayesian solution. Multivariate non-Gaussian factor-copula inference tends to be dominated by central data, and as a result, fitted models might not yield accurate model-based estimates of tail dependence parameters or reliable joint tail inference. When plots of data and likelihood analysis suggest asymmetric tail dependence, the authors propose a Bayesian approach that incorporates previous information about tail dependence to construct a prior, which can be used with the likelihood for extreme-value inference based on joint upper tails and joint lower tails. A real-world example and a simulation study show that this approach improves extreme value inference by giving more weight to the joint tails. Here we see an example of researchers applying Bayesian methods to improve inference in cases that arise in research using non-Bayesian methods.

Point patterns in geographic spaces, such as the pattern of locations of cases of an infectious disease or the coordinates of plants infected by a pest, may be interpreted as realizations of spatial point processes—stochastic processes that produce locations in their respective spaces. “Bayesian Modeling for Nonstationary Spatial Point Process via Spatial Deformations” [contribution 12] seeks to treat cases in which the potentially simplifying assumptions of isotropy and stationarity are unrealistic. The authors propose a point process analysis model that incorporates anisotropy and nonstationarity via a multidimensional Gaussian deformation process. Their approach uses Bayesian inference via MCMC (specifically, HMC) to estimate the intensity function and is shown to outperform approaches that do not permit anisotropy and nonstationarity, both for synthetic and real-world data. In the case of data on infestation of corn plants by an insect pest, the new approach identifies anisotropy that should be expected due to the way corn is planted in rows. The flexibility of Bayesian methods allows important complicating factors such as anisotropy and nonstationarity to be included in the modeling, leading to more accurate estimates of infestation levels, which in turn allow for targeted pesticide use, potentially yielding environmental and consumer health benefits.

Bayesian inference has had a major impact on how medical studies are conducted. The use of Bayesian techniques has enabled more ethical and more efficient medical studies, while simultaneously providing methods that are more reliable and harder to manipulate or “hack” than the deeply flawed Neyman–Pearson hypothesis tests involved in so many of the highly publicized, non-reproducible results in science, including medical science. “Dose Finding in Oncology Trials Guided by Ordinal Toxicity Grades Using Continuous Dose Levels” [contribution 13] is an example of applying Bayesian techniques to medical studies, specifically in first-in-humans dose-finding trials for cancer treatments. Doses for the next cohort are set by controlling the posterior probability of a patient receiving a dose above the maximum tolerated dose. The methodology made possible the first phase-1 human trial with continuous dose levels that considers grade-2 toxicity in addition to the usual binary dose-limiting toxicity, demonstrating how the adaptability of Bayesian inference continues to support innovation in medical studies while fulfilling all the ethical requirements for human trials.

There are several different common approaches to statistical inference, each with one or more underlying theories of inference. “Relative Belief Inferences from Decision Theory” [contribution 14] contains a new addition to the author’s ongoing program of developing a theory of inference that starts with a clear definition of statistical evidence—something mentioned in all theories but clearly defined in few—along with a few *desiderata*, such as reparametrization invariance. The theory is distinctly Bayesian, using Bayes’s theorem to update prior distributions with experimental data to obtain posterior distributions, from which inferences can be made, and these inferences have been shown to have certain optimality properties. The contribution to relative-belief inference theory presented in this Special Issue shows how inferences in relative belief theory can arise in a decision-theoretic context.

The authors of “A Bayesian Approach for Modeling and Forecasting Solar Photovoltaic Power Generation” [contribution 15] present data on solar power generation recorded at 74 moments each day over 19 days at a photovoltaic plant installed on a university campus. They develop a Bayesian kriging (Gaussian-process regression) scheme to use the available history of power generation at *k* recording moments per day over *N* days to predict power generation on the ${\left(N+1\right)}^{\mathrm{th}}$ day. The article adds to the variety of Bayesian techniques applied in the present Special Issue and provides a further example of how the flexibility of Bayesian techniques facilitates new analysis.

“Bayesian Assessment of Corrosion-Related Failures in Steel Pipelines” [contribution 16] presents a real-world example in which the limitations of the available historical data are mitigated by the use of expert knowledge, making it a perfect opportunity for the application of Bayesian techniques. The authors describe how they transform the knowledge of gas-distribution-pipe engineers and technicians into prior distributions and how they use these priors in Bayesian analysis to understand the roles of multiple types of corrosion in the failure of gas pipes. They also demonstrate how this analysis informs practical recommendations in both the design and maintenance phases of gas distribution networks.

Sixteen articles, of course, cannot come close to covering the immense breadth and depth of current directions in Bayesian research. However, our selection includes theoretical advances and novel practical applications, subjectivist-Bayesian and objectivist-Bayesian parametric inference, Bayesian non-parametic inference and Bayesian kriging, Bayesian model selection tools, prior elicitation, Bayesian robustness, Bayesian spatial and spatio-temporal modeling, Bayesian extreme value theory, Bayesian analysis of inference in the presence of nuisance parameters, Bayesian techniques applied to problems in research involving non-Bayesian inference, and a Bayesian philosophy-of-science approach to some of the most fundamental issues in all endeavors that involve data. Additionally, the Special Issue includes applications of Bayesian analysis in machine learning, finance, macroeconomics, genetics, epidemiology, medical trials, industry, and public policy. Like an actual photographic snapshot, our Special Issue, as a “snapshot” of the state of Bayesian inference in 2024, cannot capture the entire scope of the field but offers a glimpse of its key subjects. This snapshot provides insight into a variety of current research directions and practical applications of Bayesian inference in real-world problems.

## References

[1] Pereira C., Stern J. (1999). Evidence and Credibility: Full Bayesian Significance Test for Precise Hypotheses. Entropy.

[2] Pereira C., Stern J., Wechsler S. (2008). Can a significance test be genuinely Bayesian?. Bayesian Anal..

[3] Pereira C., Stern J. (2022). The *e*-value: A fully Bayesian significance measure for precise statistical hypotheses and its research program. São Paulo J. Math. Sci..

[4] Andrade P., Rifo L.L.R., Torres S., Torres-Avilés F. (2015). Bayesian Inference on the Memory Parameter for Gamma-Modulated Regression Models. Entropy.

[5] Cerezetti F.V., Stern J.M. (2012). Non-arbitrage in Financial Markets: A Bayesian Approach for Verification. AIP Conf. Proc..

[6] Chaiboonsri C., Wannapan S., Saosaovaphak A., Tsouris N., Vlachvei A. (2018). Economic and Business Cycle of India: Evidence from ICT Sector. Advances in Panel Data Analysis in Applied Economic Research.

[7] Chakrabarty D. (2017). A New Bayesian Test to Test for the Intractability-Countering Hypothesis. J. Am. Stat. Assoc..

[8] Chen C.W.S., Lee S. (2015). A local unit root test in mean for financial time series. J. Stat. Comput. Simul..

[9] García J.E., González-López V., Nelsen R.B. (2016). The Structure of the Class of Maximum Tsallis-Havrda-Chavat Entropy Copulas. Entropy.

[10] Maranhao V.d.L., Lauretto M.d.S., Stern J.M. (2012). FBST for Covariance Structures of Generalized Gompertz Models. AIP Conf. Proc..

[11] Kelter R. (2022). The evidence interval and the Bayesian evidence value: On a unified theory for Bayesian hypothesis testing and interval estimation. Br. J. Math. Stat. Psychol..

[12] Gannon M.A., Pereira C.A.d.B., Polpo A. (2019). Blending Bayesian and Classical Tools to Define Optimal Sample-Size-Dependent Significance Levels. Am. Stat..

[13] Pereira C.A.d.B., Nakano E.Y., Fossaluza V., Esteves L.G., Gannon M.A., Polpo A. (2017). Hypothesis Tests for Bernoulli Experiments: Ordering the Sample Space by Bayes Factors and Using Adaptive Significance Levels for Decisions. Entropy.

